# Examining the Intersection between Gender, Community Health Workers, and Vector Control Policies: A Text Mining Literature Review

**DOI:** 10.4269/ajtmh.21-0619

**Published:** 2022-01-24

**Authors:** Ana De Menezes, Ana Carolina Nunes, Denise Nacif Pimenta, Gabriela Lotta, Theresia Nkya, Morgana Martins Krieger, Brunah Schall, Clare Wenham

**Affiliations:** ^1^ Department of Geography and Environment, The London School of Economics and Political Science (LSE), London, United Kingdom;; ^2^ Getulio Vargas Foundation (FGV EAESP), São Paulo, Brazil;; ^3^ René Rachou Institute (Fiocruz Minas), Oswaldo Cruz Foundation, Belo Horizonte, Brazil;; ^4^ International Center of Insect Physiology and Ecology, Nairobi, Kenya;; ^5^ College of Health and Allied Sciences, University of Dar es Salaam-Mbeya, Dar es Salaam, Tanzania;; ^6^ Department of Health Policy, The London School of Economics and Political Science (LSE), London, United Kingdom

## Abstract

Gender intersects with healthcare systems; this is equally true for arboviral vector control efforts. However, there is as yet no comprehensive analysis as to how vector control is gendered. Hence, our objective is to provide the first thematic scoping and spatial distribution of the literature on gender, community health workers, and vector control. The authors use a systematic review approach to collect the academic literature on gender, community health workers, and vector control in Web of Science, Scopus, and PubMed (7,367 articles). After applying the exclusion criteria, 2,812 articles were analyzed using machine learning techniques: text mining and quantitative text analysis. The authors use topic modeling to assess the thematic scope of the literature and analyze the spatial distribution of themes. Our results show that the literature’s spatial scope is strongly represented by the global south as research was conducted mainly in Latin America, Africa, and Asia, places with greater incidence of vector-borne disease and with health systems, which incorporate community healthcare workers. However, there are significant spatial heterogeneities in where and how research is conducted. The topic analysis reveals that the literature predominantly considers issues of sex (e.g., pregnancy) and gender as it relates motherhood. Gendered considerations occur upon implementation of vector control policies, rather than being mainstreamed into their development and delivery. There is a need to deepen the analysis to allow for gendered aspects to be understood beyond binary sex differences and/or reproductive health.

## INTRODUCTION

Arboviruses and vector control remains a key public health issue for many locations in the Global South: Brazil has recently suffered from the largest dengue outbreak to date; similar trends can be found in Angola, Republic of the Congo, and Democratic Republic of the Congo.^1^ There has been increasing consideration of the interrelation between gender and vector control—taking into account the definition of gender as a social construct that determines the behavior and actions of men, women, and nonbinary individuals.^2^ This interrelation appears, for example, in discussions on how sociocultural gender norms affect both the exposure to arboviruses and the capacity to seek treatment.^3^ Women have been seen not only as targets of vector control policies, as in the case of avoiding arboviruses infections during pregnancy,^4^^,^^5^ but also as active agents in the fight against these infectious diseases within their communities and households.^6^ These roles may include women as enforcers of treatment adherence, firsthand caregivers, and promoter of usage of intervention tools, among others.

Women are also involved as unpaid or underpaid community health workers (CHWs)^7^^,^^8^ who keep communities informed, help identify disease, and distribute medications or interventions that help prevent infections. These roles in vector control programs traditionally have followed well-established cultural gender norms:^3^^,^^9^ activities linked to community and domestic care are mostly developed by women (e.g., clearing stored water in communities and health education), whereas men tend to be more involved in vector control efforts reliant on physical strength (e.g., fogging), implementation of interventions and decision-making.

Community health workers are a bridge between community needs and basic healthcare access. From India’s National Village Health Guides Scheme to Kenya’s Community Health Volunteer Program, CHWs have a fundamental role in identifying the most vulnerable individuals to infectious diseases as well as facilitating the provision of treatment to families in their community. They provide this service while recognizing that the various CHWs programs are specific to their social, cultural, and economic contexts.

It’s through CHW programs that women are mostly involved in vector control strategies around the globe, although these strategies may be found in several configurations, with more or less participation of CHWs. The introduction of CHW programs takes advantage of this gendered labor division as they allocate care work inside communities primarily to women and thus reinforce gender inequalities in households and healthcare workforce.^7^^,^^10^ On the other hand, there is evidence that CHW strategies may reduce gender differences in the implementation of community health services,^11^ increasing the coverage of treatment,^12^ mobility, and social recognition from women in their communities.^13^

Although gender analysis has been frequently used to analyze the role of CHW, the same cannot be said for vector control programs. Gender considerations in this area not only enrich the analysis but add to our understanding of the mechanisms of program implementation. This research therefore seeks to understand how gender norms have been portrayed in the literature about vector control policies that mobilize CHW strategies.

## METHODS

### Search strategy and selection criteria.

The academic literature search on gender, vector control, and CHWs followed a systematic framework based on PRISMA. We collected all articles in three databases (Web of Science, Scopus, and PubMed) that used our specified keywords in the title, abstract, or keywords. The search was conducted in English, Spanish, and Portuguese. The keywords were part of three distinct search strings that focused on 1) gender and vector control, 2) gender and CHWs, and 3) CHWs and vector control. We considered variations in the categorization of gender, CHWs, and vector control to encompass institutional arrangements across countries (for full description of search words, see Supplemental Information). These searches yielded 7,367 articles. After eliminating the duplicates, the final result for these databases was 6,974 documents.

The next phase consisted of manually applying the exclusion criteria to the documents. These criteria were 1) documents need to comprise elements of vector control policies or community health strategies; 2) if the article is a case study, it needs to be related to arboviruses or malaria; and 3) documents should contain abstracts. These criteria ensured that we only kept documents that were either original research articles, opinion articles, or review articles, excluding other document types such as book chapters, errata, editorials, conference abstracts, or conference papers. This screening resulted in 2,812 documents.

### Data analysis.

We used text mining to analyze 2,812 documents related to gender, vector control, and CHWs. These data were analyzed using the Quanteda package in R and Latent Dirichlet Allocation (LDA) modeling in Python.^14^^–^^16^

The approach was 2-fold. First, we sought to understand the spatial distribution of the literature and if there are any clusters of knowledge across different regions. To map these patterns, we extracted and geolocated all names referring to places, countries, or regions in each abstract and title. To identify and geolocate places, we used a geographical dictionary from the newsmap package, which is able to select distinct countries from a text as well as determine what is the predominant country/region in a specific abstract.

Second, a correlated topic model^17^ was used to assess the main themes in the literature. We explored the thematic scope of our target literature to identify the various topics in it and how they interact with one another. This was achieved by using a topic modeling algorithm, which consists of unsupervised natural language processing used to find the hidden thematic structure in the text. This was done by using a probabilistic modeling approach called LDA.

## RESULTS

### Spatial distribution.

**Figure 1.**
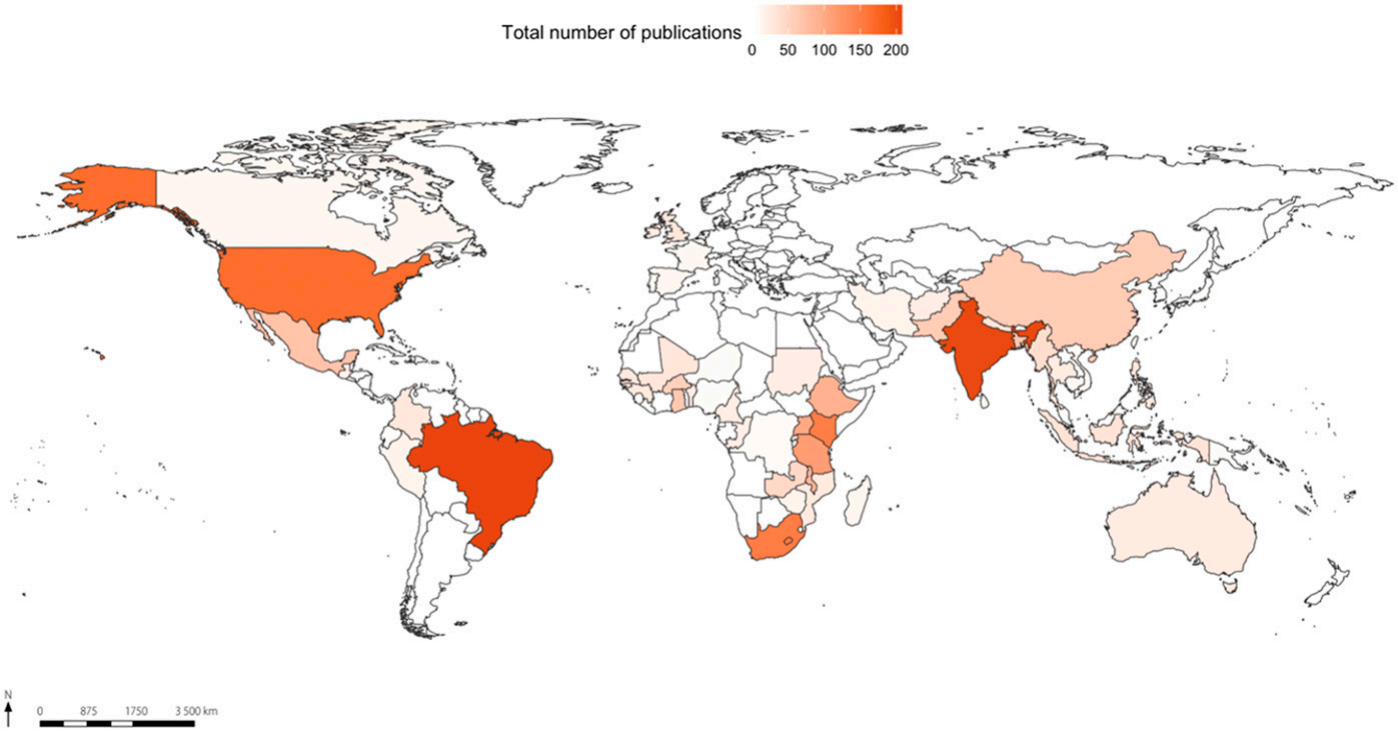
Map of the spatial distribution of 2,812 publications related to gender, community health workers, and vector control. This figure appears in color at www.ajtmh.org.

Our search of the literature yielded a final dataset, or corpus, of 2,812 abstracts from the years 1932–2020. The pooled dataset containing gender, vector control policies, and community health work information (Figure 1) reveals that the majority of articles refers to research in some spatial clusters, particularly eastern and southern Africa, southeast Asia, and few countries in Latin America.

**Figure 2.**
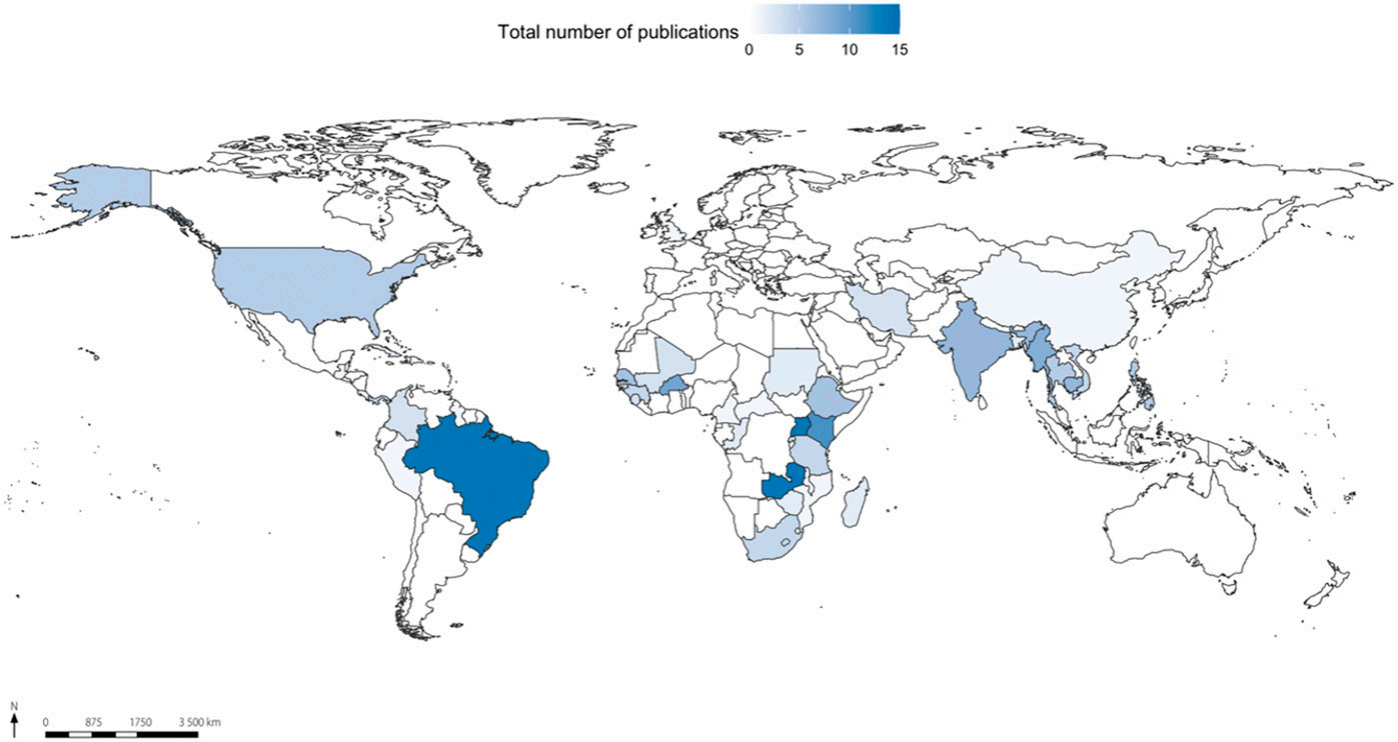
Map of the spatial distribution of 188 publications related to the search string “vector control and community health workers.” This figure appears in color at www.ajtmh.org.

A more detailed analysis (Figure 2) on the distribution of CHWs and vector control policies research shows that 10 countries contain the highest number of publications: Uganda (14), Zambia (14), Brazil (14), Kenya (11), Burkina Faso (9), Myanmar (8), India (7), Cambodia (7), Ethiopia (6), and Senegal (6). This distribution corroborates the pattern found in the distribution of the general dataset (Figure 1).

**Figure 3.**
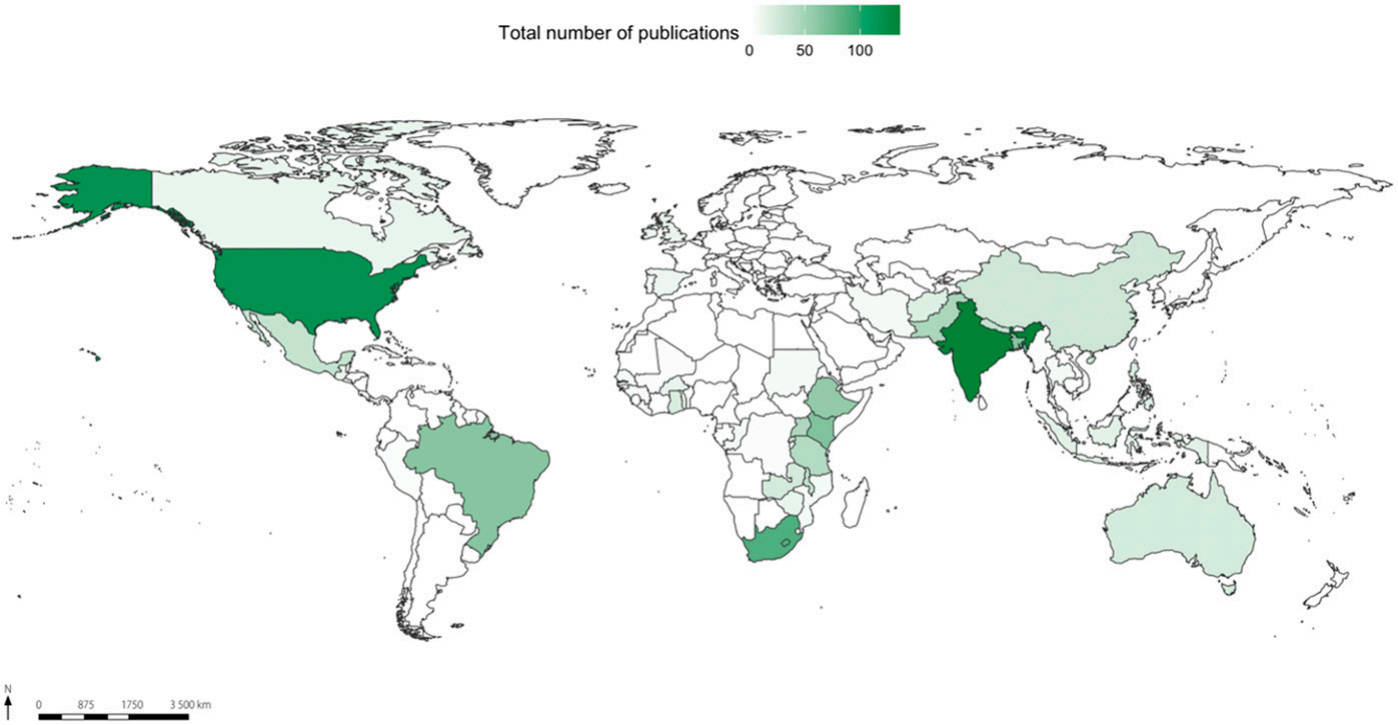
Map of the spatial distribution of 1,271 publications related to the search string “community health workers and gender.” This figure appears in color at www.ajtmh.org.

The aforementioned pattern is intensified when considering CHWs and gender (Figure 3). In this context, some countries represent a concentration of research. From the 1,271 documents analyzed, the most representative countries are India (126), United States (109), South Africa (84), Kenya (64), Bangladesh (63), Brazil (62), Ethiopia (56), Uganda (44), Pakistan (44), and Tanzania (38).

**Figure 4.**
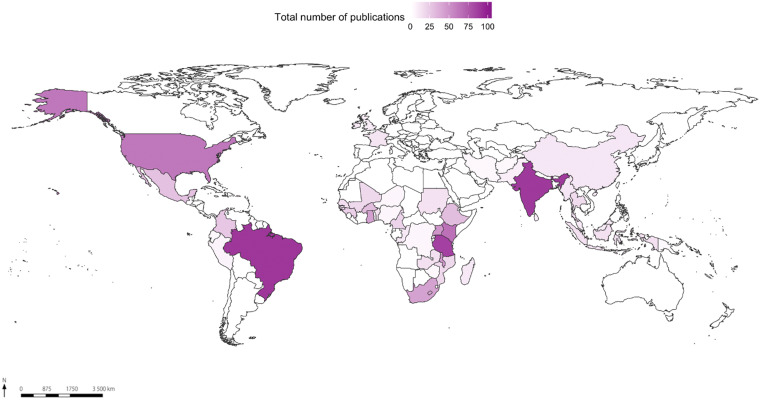
Map of the spatial distribution of 1,256 publications related to the search string “gender and vector control.” This figure appears in color at www.ajtmh.org.

The scenario repeats itself in the third string analyzed: gender and vector control policies. Here, 1,256 documents were analyzed in which the concentration pattern is represented by the most common countries (Figure 4). The 10 countries with the highest volume of documents are Brazil (99), India (90), Tanzania (86), Malawi (76), Kenya (72), United States (64), South Africa (52), Uganda (51), Ghana (47), and Burkina Faso (36).

### Topics.

**Figure 5.**
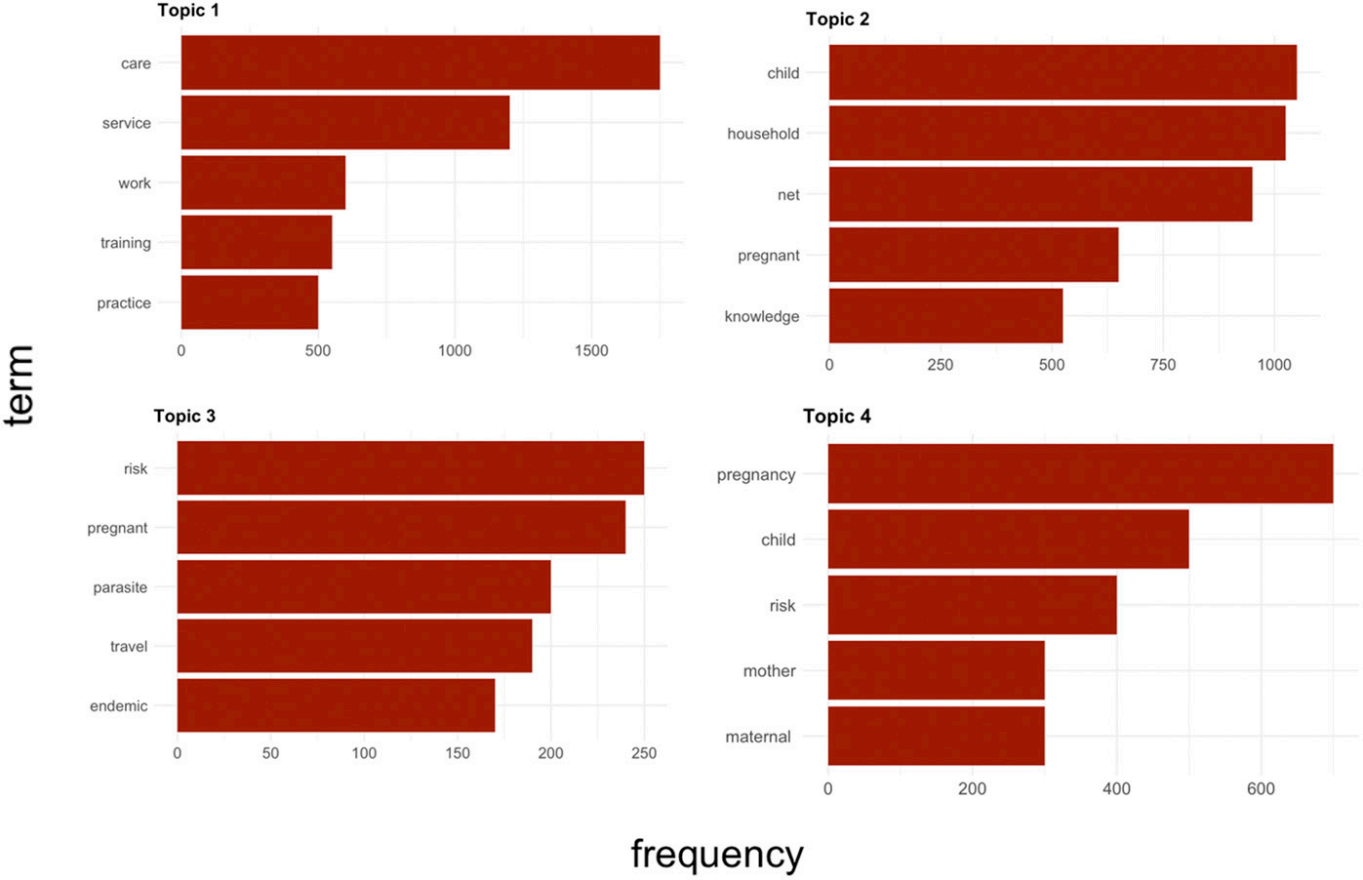
Most relevant terms of four identified clusters (topics). This figure appears in color at www.ajtmh.org.

Figure 5 shows that the thematic scope can be aggregated into four groups denoting the proximity in meaning of the words in each group. It also shows the frequency of key words in the documents. Using this approach, we can identify the relative importance of some words in the literature of vector control and gender. Here, we see that words connected to reproductive health are the most frequent; the most common words are “pregnancy,” “child,” and “maternal.” Words that denote care activities are also frequent, including “care” and “service.” This clustering indicates that the literature is highly concentrated in themes in which gender is viewed as a reproductive health issue within research on vector control policies.

## DISCUSSION

The results show that the geographical distribution of research on vector control policies and gender is concentrated in some regions, particularly west Africa, southeast Asia, and some countries of Latin America. It also indicates that gender is predominantly seen in terms of reproductive health.

These results reinforce the importance of analyzing gender in health policies beyond the “targeting” of women for better vector control. Furthermore, they help to put the focus on how gender relations are expressed and reinforced through health policies.^12^ Importantly, these results also highlight a lacuna of evidence in the role of gender in vector control, and this should be considered seriously for future research to understand the inner workings of effective, sustainable programs. Other themes that could be relevant to the discussion of gender are only rarely present, such as unpaid labor and decision-making. These findings reveal an important gap in the global literature concerning the role of gender norms when dealing with CHWs and vector control policies.

The data predominantly considers issues related to biological sex reproductive health, in as much as it does gender. That is, the key words that appear consistently are pregnancy, and the associated gendered reproduction of mother and motherhood. In doing so, this shows that in many studies, women are reduced to their reproductive function (and we use women here to include nonbinary people who bear children), and in doing so, compounds that role and the understanding of gendered social norms in this way.^18^^,^^19^ This is important to highlight for a number of reasons: first, when research considers women, it considers them as pregnant or mothers rather than a consideration of gender in societies; the gendered norms, which dominate social, economic, and civic life, which ascribe certain tasks to women, tacitly, and often, which invisibilize informal labor within households and communities, which women might perform.^20^^,^^21^ For example, women are more likely to care for children, not for biological reasons, but for social attribution of responsibility.^22^ Thus, efforts for vector control associated with children are deeply connected to the role of women or mothers—including interacting with health services, eliminating breeding sites, and taking care of children when they are infected with disease. These gendered norms extend into society and into vector control—those who are most likely to absorb the burden of civic or household cleaning to reduce standing water and reservoirs are most likely to be women, as we know that women are more likely to work in community activities and voluntary work. This informal labor is the bedrock of much of the content provided in the papers analyzed, from development to education, from nets to care, but the explicit gender considerations of this are alarmingly absent.

Moreover, women are disproportionately used as community healthcare workers, and thus the labor involved in vector control is not only gendered within households, but in formal arrangements too.^23^ Scholars show how CHWs are usually used on precarious contracts, receive low salaries, are less trained, have received less personal protective equipment and resources during COVID, and many times have to assume responsibilities viewed as an extension of the labor at home.^7^^,^^8^^,^^10^ Also reflecting gender norms, women working in the field of care are expected to be attentive and emotionally involved—what may reflect, among CHWs, under pressure to provide care to their communities even when they are not paid for it.^13^ The fact that they are part of the community for whom they work also exposes them to multiple vulnerable situations. For example, the lack of privacy, the need to work extra hours and over weekends, and even the lack of legitimacy to enforce some health measures.^8^^,^^24^

Where we do find gender discussions in our data, these appear in the implementation and/or end user of vector control. However, there is a notable absence in discussion of gender within decision-making processes or policy formulation of vector control. This is not so much a question of gender parity in decision-making, while this is important, it would not necessarily feature in literature review, but rather a concern that the gendered considerations have not formed part of policy development, such as to gender mainstream, or to create gender transformative policy.^25^ As part of the sustainable development goals, gender equity is a key concern, and there have been many efforts to push for gender-mainstreamed policy across the global health space, most notably in discussion of health systems, HIV/AIDS control, and more recently in COVID-19. It is a surprise that this has not been a feature of vector control to date, given the inherent gendered norms associated with delivery.^26^

Where we do see increased consideration of gender within vector control, the thematic scope reveals that this has mostly emerged from global institutions, such as the sustainable development goals, WHO, and the broader UN system. This is notably in contrast to national policies in which such gender considerations are largely absent. In part, this reflects increasing global gender norms, which see gender mainstreaming and gender equity as paramount to all activity, and gender in all policies as promoted by WHO, and the UN system.^27^^–^^29^ Yet, vector control policy and efforts are determined at the national level, and thus global championing of gender will not necessarily lead to meaningfully gender-sensitive policy. Future research must analyze this intersection and seek pathways to domestic engagement with gender in vector control.

To strengthen vector control efforts around the globe, it is important that the literature updates its gender lens regarding vector control programs advancing toward a more comprehensive approach about the role played by gender norms in the fight against arboviruses. The analysis presented faced some limitations related to the ability of artificial intelligence to identify all duplicates and articles with no abstract; besides, the trade-off of having to choose between analyzing each article in depth or accessing the whole sample due to the large volume of articles found. Finally, our search strategy only addresses documents in which CHW-related strategies in vector control programs are studied; this conditions geographical limitations in the analysis.

As a future agenda, it is suggested to analyze how different vector control and CHW strategies (whether voluntary or paid, governmental or nongovernmental, nationally or internationally implemented) might have different impacts on gender norms, especially regarding topics such as sexual and reproductive rights, access to income, and to primary healthcare.

## Supplemental Material


Supplemental materials
