# Supplementary material for: Examining the Intersection between Gender, Community Health Workers, and Vector Control Policies: A Text Mining Literature Review
Source: Am J Trop Med Hyg. 2022 Jan 24;106(3):768–74. doi: 10.4269/ajtmh.21-0619 (PMC8922516; doi:10.4269/ajtmh.21-0619)
Supplement: Supplementary file 1 [file tpmd210619.SD1.pdf]

Examining the intersection between gender, community health workers and vector control policies: a text mining literature review

Ana De Menezes[1], Ana Carolina Nunes[2], Denise Nacif Pimenta[3], Gabriela Lotta[2], Theresia E. Nkya[5], Morgana G. Martins Krieger[2], Brunah Schall[3], Clare Wenham[8*]

Suplementary information

Search Strings

Examining the intersection between gender, community health workers and vector control policies: a text mining literature review<br>Search Strings						
Database	Database category	String category	Amount	Type of documents (non-exclusive)	String details	library
Web of Science	All databases	Gender & vector control	2,068	ARTICLE (1,984); OTHER (928); REVIEW (256); ABSTRACT (110); MEETING (99); CLINICAL TRIAL (96); UNSPECIFIED (49); REPORT (44); EDITORIAL (38); LETTER (17); BOOK (10); EARLY ACCESS (7); NEWS (5); REFERENCE MATERIAL (4); CORRECTION (4); BIOGRAPHY (1); PATENT (1);	TS = ((gender* OR wom*) AND (((vector OR dengue OR zika OR mosquito OR malaria OR aedes OR arbovir* OR chikungunya) NEAR/2 (control OR management OR program* OR strateg*)) OR "vector control"))	Endnote online

				CASE REPORT (1)		
Web of Science	All databases	Vector control & community health workers	373	ARTICLE (354); OTHER (199); CLINICAL TRIAL (35); REVIEW (26); ABSTRACT (20); MEETING (18); UNSPECIFIED (8); EDITORIAL (4); BOOK (4); LETTER (2)	TS = ((((vector OR dengue OR zika OR mosquito OR *malaria OR aedes OR arbovir* OR chikungunya*) NEAR/2 (control OR management OR program* OR strateg*)) OR "vector control") AND ("community health worker*" OR "community worker*" OR "community health practitioner*" OR *("community health" NEAR/2 worker) OR "vector control agent"*))	Endnote online
Web of Science	All databases	Community health worker & gender	2411	ARTICLE (2,354); OTHER (1,169); CLINICAL TRIAL (290); REVIEW (156); ABSTRACT (67); MEETING (51); UNSPECIFIED (47); EDITORIAL (24); EARLY ACCESS (20); CASE REPORT (12); LETTER (7); BOOK (3); NEWS (2); BIOGRAPHY (1); LEGISLATION (1); REFERENCE MATERIAL (1);	TS = ((gender* OR wom*) AND ("community health worker*" OR "community worker*" OR "community health practitioner*" OR ("community health" NEAR/2 worker) OR "vector control agent"))	Endnote online

				BIBLIOGRAPHY (1); REPORT (1)		
Web of Science	All databases	COVID & gender & vector control	8	ARTICLE (7); OTHER (2); BOOK (1); CASE REPORT (1); REVIEW (1)	TS = (((COVID* OR corona*) AND (gender* OR wom*)AND (((vector OR dengue OR zika OR mosquito OR malaria OR aedes OR arbovir* OR chikungunya) NEAR/2 (control OR management OR program* OR strateg*)) OR "vector control")))	Endnote online
Web of Science	All databases	COVID & gender & community health workers	17	ARTICLE (17); OTHER (10); REVIEW (3) ; CLINICAL TRIAL (2); EARLY ACCESS (1); UNSPECIFIED (1)	TS = ((COVID* OR corona*) AND (gender* OR wom*) AND (“community health worker*” OR “community worker*” OR “community health practitioner*” OR (“community health” NEAR/2 worker) OR “vector control agent”))	Endnote online
Web of Science	All databases	COVID & community health worker & vector control	0	0	TS = (((COVID* OR corona*) AND (((vector OR dengue OR zika OR mosquito OR malaria OR aedes OR arbovir* OR chikungunya) NEAR/2 (control OR management OR program* OR strateg*)) OR "vector control") AND (“community health worker*” OR “community worker*” OR “community health practitioner*” OR (“community health” NEAR/2 worker) OR “vector control agent”)))	Endnote online
Scopus	All databases	Gender & vector control	361	Article (276); Review (52); Book Chapter (7); Note (7); Conference Paper (6); Editorial (6); Letter (5); Short Survey (1); Undefined (1)	TITLE-ABS-KEY((gender* OR wom*) AND (((vector OR dengue OR zika OR mosquito OR malaria OR aedes OR arbovir* OR chikungunya) NEAR/2 (control OR management OR program* OR strateg*)) OR "vector control"))	Endnote online
Scopus	All databases	Vector control & community health workers	84	Article (73); Review (8); Book Chapter (2); Conference	TITLE-ABS-KEY((((vector OR dengue OR zika OR mosquito OR malaria OR aedes OR arbovir* OR chikungunya) NEAR/2 (control OR management OR program* OR strateg*)) OR "vector control")	Endnote online

				Paper (1)	AND (“community health worker*” OR “community worker*” OR “community health practitioner*” OR (“community health” NEAR/2 worker) OR “vector control agent”))	
Scopus	All database s	Communit y health worker & gender	1,628	Article (1,480); Review (92); Conference Paper (20); Book Chapter (10); Note (8); Book (4); Letter (4); Short Survey (4); Conference Review (3); Editorial (3)	TITLE-ABS-KEY((gender* OR wom*) AND (“community health worker*” OR “community worker*” OR “community health practitioner*” OR (“community health” NEAR/2 worker) OR “vector control agent”))	Endnot e online
Scopus	All database s	COVID & gender & vector control	1	Short Survey (1)	TITLE-ABS-KEY(((COVID* OR corona*) AND (gender* OR wom*)AND (((vector OR dengue OR zika OR mosquito OR malaria OR aedes OR arbovir* OR chikungunya) NEAR/2 (control OR management OR program* OR strateg*)) OR "vector control")))	Endnot e online
Scopus	All database s	COVID & gender & communit y health workers	8	Article (5); Review (2); Short Survey (1)	TITLE-ABS-KEY((COVID* OR corona*) AND (gender* OR wom*) AND (“community health worker*” OR “community worker*” OR “community health practitioner*” OR (“community health” NEAR/2 worker) OR “vector control agent”))	Endnot e online
Scopus	All database s	COVID & communit y health worker & vector control	0		TITLE-ABS-KEY(((COVID* OR corona*) AND (((vector OR dengue OR zika OR mosquito OR malaria OR aedes OR arbovir* OR chikungunya) NEAR/2 (control OR management OR program* OR strateg*)) OR "vector control") AND (“community health worker*” OR “community worker*” OR “community health practitioner*” OR (“community health” NEAR/2 worker) OR	Endnot e online

					“vector control agent”)))	
PUBMED	All databases	Gender & vector control	83		TITLE/ABSTRACT((gender* OR wom*) AND (((vector OR dengue OR zika OR mosquito OR malaria OR aedes OR arbovir* OR chikungunya) NEAR/2 (control OR management OR program* OR strateg*)) OR "vector control"))	csv
PUBMED	All databases	Vector control & community health workers	56		TITLE/ABSTRACT((((vector OR dengue OR zika OR mosquito OR malaria OR aedes OR arbovir* OR chikungunya) NEAR/2 (control OR management OR program* OR strateg*)) OR "vector control") AND (“community health worker*” OR “community worker*” OR “community health practitioner*” OR (“community health” NEAR/2 worker) OR “vector control agent”))	csv
PUBMED	All databases	Community health worker & gender	269		TITLE/ABSTRACT((gender* OR wom*) AND (“community health worker*” OR “community worker*” OR “community health practitioner*” OR (“community health” NEAR/2 worker) OR “vector control agent”))	csv
PUBMED	All databases	COVID & gender & vector control	0		TITLE/ABSTRACT(((COVID* OR corona*) AND (gender* OR wom*)AND (((vector OR dengue OR zika OR mosquito OR malaria OR aedes OR arbovir* OR chikungunya) NEAR/2 (control OR management OR program* OR strateg*)) OR "vector control")))	csv
PUBMED	All databases	COVID & gender & community health workers	0		TITLE/ABSTRACT((COVID* OR corona*) AND (gender* OR wom*) AND (“community health worker*” OR “community worker*” OR “community health practitioner*” OR (“community health” NEAR/2 worker) OR “vector control agent”))	csv
PUBMED	All	COVID &	0		TITLE/ABSTRACT(((COVID* OR corona*) AND (((vector OR	csv

D	databases	community health worker & vector control			dengue OR zika OR mosquito OR malaria OR aedes OR arbovir* OR chikungunya) NEAR/2 (control OR management OR program* OR strateg*)) OR "vector control") AND (“community health worker*” OR “community worker*” OR “community health practitioner*” OR (“community health” NEAR/2 worker) OR “vector control agent”)))	
